# Complete Genome Sequence of the Freshwater Colorless Sulfur Bacterium *Beggiatoa leptomitiformis* Neotype Strain D-402^T^

**DOI:** 10.1128/genomeA.01436-15

**Published:** 2015-12-10

**Authors:** Alexey Fomenkov, Tamas Vincze, Margarita Y. Grabovich, Galina Dubinina, Maria Orlova, Elena Belousova, Richard J. Roberts

**Affiliations:** a New England BioLabs, Ipswich, Massachusetts, USA; b Department of Biology, Voronezh State University, Voronezh, Russia; c Winogradsky Institute of Microbiology, Russian Academy of Sciences, Moscow, Russia

## Abstract

In this report, we announce the availability of a complete closed genome sequence and methylome analysis of *Beggiatoa leptomitiformis* neotype strain D-402^T^ (DSM 14946, UNIQEM U 779).

## GENOME ANNOUNCEMENT

At the end of the 19th century, Sergei Winogradsky introduced the concept of chemolithotrophy when he first reported on organisms gaining energy exclusively from the oxidation of inorganic compounds (1). Members of the bacterial family *Beggiatoaceae* have gained much attention due to their ability to oxidize sulfide to elemental sulfur, which they deposit intracellularly in the form of small globules or droplets. However, due to the difficulties of isolating, purifying, and growing this bacterium *in vivo*, only a few draft genome sequences have been assembled (accession no. NZ_AHMA00000000.1, GCA_000170695.1, and GCA_000170715.1).

In this report, we announce the availability of a complete closed genome sequence of *Beggiatoa leptomitiformis* neotype strain D-402^T^ (DSM 14946, UNIQEM U 779). This strain was previously described based on its morphological and biochemical characteristics (2–6).

The genome was sequenced using the Pacific Biosciences (PacBio) RSII sequencing platform (7). Briefly, SMRTbell libraries were constructed from a genomic DNA sample sheared to an average size of ~10 to 20 kb using the G-tubes protocol (Covaris, Woburn, MA, USA), additionally purified using the PowerClean DNA clean-up kit (MoBio Laboratories, Inc., Carlsbad, CA), end repaired, and ligated to hairpin adapters. Incompletely formed SMRTbell templates were digested with a combination of exonuclease III and exonuclease VII (New England BioLabs, Ipswich, MA, USA). Genomic DNA fragments and SMRTbell library qualification and quantification were performed using a Qubit fluorimeter (Invitrogen, Eugene, OR) and 2100 Bioanalyzer (Agilent Technologies, Santa Clara, CA). Two SMRTbell 10- and 20-kb libraries were prepared according to the modified 10- and 20-kb PacBio sample preparation protocols and sequenced using C2 and C4 chemistry on six single-molecule real-time (SMRT) cells, with a 180-min collection protocol. Sequencing reads were processed, mapped, and assembled by the Pacific Biosciences SMRT Analysis pipeline using the HGAP3 protocol and polished using Quiver (8) to give a fully closed genome with 422× coverage. The genome size was 4,265,296 bp, and the plasmid was 6,185 bp, which together contain a total of 3,636 genes. The assembled sequences were annotated with Rapid Annotations using Subsystems Technology (RAST) (9) and the NCBI Prokaryotic Genomes Annotation Pipeline (PGAP).

Epigenetic modification at each nucleotide position was measured as kinetic variations (KVs) in the nucleotide incorporation rates, and methylated motifs were deduced from the KV data (10–12). Thirteen DNA methyltransferase recognition motifs corresponding to one m4C and nine m6A modifications were detected by direct single-molecule real-time (SMRT) sequencing, and an additional three m5C motifs were detected in Tet2-treated DNA. Matching of motifs with methyltransferase genes was carried out, and the results are shown in Table 1. They have also been deposited in REBASE (13).

**TABLE 1:** Summary of methyltransferases identified in *B. leptomitiformis* neotype strain D-402^T^

Motif^*a*^	Assigned or predicted	Methylation type	Restriction modification type
Direct detection			
GATC	M.Ble402I	m6A	II
GRAGC**A**G	M.Ble402II	m6A	II
SAG**C**TS	M.Ble402III	m4C	II
AC**A**YNNNNNR**T**GT		m6A	II
CA**A**YNNNNR**T**TG	S.Ble402ORFBP	m6A	I
C**A**GNNNNNR**T**AAT	S.Ble402ORFQP	m6A	I
CATCH**A**G		m6A	II
CGG**A**G		m6A	III
CGGTC**A**		m6A	II
DCTGG**A**TD		m6A	II
GGCTG**A**		m6A	II
GTTGN**A**G		m6A	II
TCG**A**		m6A	II
5-mC oxidation by Tet2			
GGHCC = GGNCC	M.Ble402ORFDP	5 mC	II
CCDGG = CCNGG	M.Ble402ORFLP	5 mC	II
GGCCNB = GGCC	M.Ble402ORFTP	5 mC	II

a Modified bases are highlighted in bold.

### Nucleotide sequence accession numbers.

The complete genome and plasmid sequences of the *B. leptomitiformis* neotype strain D-402^T^ are available in GenBank under the accession numbers CP012373 and CP012374, respectively.
