# Correction for Fomenkov et al., “Complete Genome Sequence of the Freshwater Colorless Sulfur Bacterium Beggiatoa leptomitoformis Neotype Strain D-402^T^”

**DOI:** 10.1128/genomeA.00312-18

**Published:** 2018-04-19

**Authors:** Alexey Fomenkov, Tamas Vincze, Margarita Y. Grabovich, Galina Dubinina, Maria Orlova, Elena Belousova, Richard J. Roberts

**Affiliations:** a New England BioLabs, Ipswich, Massachusetts, USA; b Department of Biology, Voronezh State University, Voronezh, Russia; c Winogradsky Institute of Microbiology, Russian Academy of Sciences, Moscow, Russia

## AUTHOR CORRECTION

Volume 3, no. 6, e01436-15, 2015, https://doi.org/10.1128/genomeA.01436-15. Pages 1 and 2: The article title should read as given above, and the species name should be identified throughout as leptomitoformis.

Page 2: The sequence deposited as CP012374, previously reported as a plasmid, is in fact the PacBio internal sequencing control probe. This record was removed by database staff, because it was determined to contain significant vector contamination.

